# Identification of key genes to predict response to chemoradiotherapy and prognosis in esophageal squamous cell carcinoma

**DOI:** 10.3389/fmolb.2024.1512715

**Published:** 2024-11-20

**Authors:** Yingying Cui, Jing Wen, Jianhua Fu, Changsen Leng

**Affiliations:** ^1^ State Key Laboratory of Oncology in South China, Guangdong Provincial Clinical Research Center for Cancer, Sun Yat-sen University Cancer Center, Guangzhou, China; ^2^ Department of Hematologic Oncology, Sun Yat-sen University Cancer Center, Guangzhou, China; ^3^ Guangdong Esophageal Cancer Institute, Guangzhou, China; ^4^ Department of Thoracic Surgery, Sun Yat-sen University Cancer Center, Guangzhou, China

**Keywords:** esophageal squamous cell carcinoma, chemoradiotherapy sensitivity, neoadjuvant chemoradiotherapy, pathological complete response, immune microenvironment

## Abstract

**Background:**

Chemoradiotherapy is a crucial treatment modality for esophageal squamous cell carcinoma (ESCC). This study aimed to identify chemoradiotherapy sensitivity-related genes and analyze their prognostic value and potential associations with the tumor microenvironment in ESCC.

**Methods:**

Utilizing the Gene Expression Omnibus database, we identified differentially expressed genes between ESCC patients who achieved complete and incomplete pathological responses following chemoradiotherapy. Prognostic genes were then screened, and key genes associated with chemoradiotherapy sensitivity were determined using random survival forest analysis. We examined the relationships between key genes, infiltrating immune cells, and immunoregulatory genes. Additionally, drug sensitivity and enrichment analyses were conducted to assess the impact of key genes on chemotherapy responses and signaling pathways. A prognostic nomogram for ESCC was developed incorporating key genes, and its effectiveness was evaluated. Genome-wide association study data were employed to investigate chromosomal pathogenic regions associated with key genes.

**Results:**

Three key genes including *ATF2*, *SLC27A5*, and *ALOXE3* were identified. These genes can predict the sensitivity of ESCC patients to neoadjuvant chemoradiotherapy and hold significant clinical relevance in prognostication. These genes were also found to be significantly correlated with certain immune cells and immunoregulatory genes within the tumor microenvironment and were involved in critical tumor-related signaling pathways, including the epithelial-mesenchymal transition and P53 pathways. A nomogram was established to predict the prognosis of ESCC by integrating key genes with clinical stages, demonstrating favorable predictability and reliability.

**Conclusion:**

This study identified three key genes that predict chemoradiotherapy sensitivity and prognosis and are involved in multiple tumor-related biological processes in ESCC. These findings provide predictive biomarkers for chemoradiotherapy response and support the development of individualized treatment strategies for ESCC patients.

## Introduction

Esophageal cancer ranks as the seventh most common malignant tumor globally, with approximately 604,100 new cases diagnosed annually (Sung et al., 2021). Histopathologically, it is primarily classified into esophageal squamous cell carcinoma (ESCC) and esophageal adenocarcinoma, each differing markedly in pathogenesis, biological behavior, treatment, and prognosis. ESCC, accounting for about 85% of esophageal cancers, is predominantly found in East Asia and Africa (He et al., 2021). This type is highly invasive, and symptoms such as dysphagia often manifest in the disease’s late stages, leading to a dismal prognosis with a five-year survival rate between 15% and 25% (Shi et al., 2022).

At diagnosis, nearly 50% of patients exhibit tumor invasion beyond the primary lesion’s local area, with 70%–80% presenting regional lymph node metastasis. Locally advanced ESCC is defined as stage T2-4 or N1-3 with M0 (Thakur et al., 2021; Puhr et al., 2023). The standard treatment for this stage is neoadjuvant chemoradiotherapy (NCRT) followed by surgical resection. In the CROSS trial, the NCRT group achieved a significantly higher R0 resection rate (92% vs. 69%), negative lymph node resection rates (31% vs. 75%), and improved overall survival (OS, 49.4 vs. 24 months) compared to the surgery-only group in treating locally advanced ESCC (van Hagen et al., 2012). The NEOCRTEC5010 study, involving 451 patients, demonstrated that NCRT significantly enhanced the five-year OS rate (from 49.1% to 59.9%) over surgery alone (Yang et al., 2021a). Chen et al. further validated the superiority of NCRT over neoadjuvant chemotherapy followed by surgery, showing higher pathological complete response (pCR) rates, negative lymph node resection rates, and reduced mortality due to tumor progression or recurrence in the NCRT group (Wang et al., 2021). Approximately 20%–40% of patients with locally advanced esophageal cancer achieve pCR following NCRT (van Hagen et al., 2012; Yang et al., 2021a; Wang et al., 2021). pCR is closely associated with extended OS and reduced rates of distant recurrence (Noordman et al., 2018; Hirata et al., 2021). For ESCC patients achieving pCR after NCRT, the need for esophagectomy and treatment strategies should be reassessed (Noordman et al., 2018). Furthermore, given the poor prognosis of advanced ESCC, predicting patient outcomes from chemoradiotherapy in advance can inform treatment planning (Hirata et al., 2021). Thus, identifying sensitive, specific, and accurate biomarkers to forecast ESCC patients’ responses to chemoradiotherapy, especially their pCR status, is imperative. Previous studies suggested using clinical remission or imaging techniques to predict ESCC pCR post-NCRT (Squires et al., 2022; Liu et al., 2016). However, it remains unclear who benefits most from NCRT or chemoradiotherapy among ESCC patients.

In this study, we investigated the differentially expressed genes (DEGs) between patients who achieved pCR and did not achieve pCR (npCR) following NCRT, and screened key genes associated with the prognosis of ESCC. Subsequently, we assessed the relationships between these key genes and ESCC-related genes, infiltrating immune cells, and chemotherapy sensitivity. This analysis is intended to more accurately predict chemoradiotherapy sensitivity and prognosis, thereby enhancing treatment strategies for ESCC patients.

## Methods

### Data source and preprocessing

According to the 6th edition of the American Joint Committee on Cancer TNM staging system, the Guangzhou cohort included patients with ESCC staged IIb-III who underwent NCRT prior to surgery from September 2007 to March 2012. The RNA-seq data for these patients are accessible in the Gene Expression Omnibus (GEO) database under accession number GSE45670. We monitored these patients until July 2023, selecting those who survived over 3 months after treatment to assess their survival outcomes. Another cohort, the Beijing cohort (GSE53624), comprised tumor and adjacent normal tissues from 119 ESCC patients. The Series Matrix Files for GSE45670 and GSE53624 were based on annotation platforms GPL570 and GPL18109, respectively.

We retrospectively included the real-world patients with stage II-III ESCC from Sun Yat-sen University Cancer Center. Tissue samples and pathological results of these patients were obtained by endoscopic biopsy prior to treatment. After pathological diagnosis, they received NCRT combined with esophagectomy. This study was approved by the Ethics Committee of the Sun Yat-sen University Cancer Center and conducted in accordance with the local legislation and institutional requirements.

### Identification of key chemoradiotherapy sensitivity-related genes

The “limma” package (Ritchie et al., 2015) was utilized to identify DEGs between pCR and npCR patients, while univariate Cox regression analysis determined genes associated with survival. We then identified genes that were highly expressed in pCR patients and suggested a favorable prognosis, and those underexpressed in pCR patients and indicated a poorer prognosis. The random survival forest algorithm executed using the “randomForestSRC” package, selected genes based on their prognostic importance. Genes with a relative importance exceeding 0.7 were classified as key genes related to chemoradiotherapy sensitivity.

### Analysis of immune cell infiltration in ESCC

Using the gene expression of each patient, the CIBERSORT deconvolution algorithm estimated the relative proportions of 22 immune cell types, including B cell subsets, T cell subsets, NK cells, and macrophages (Newman et al., 2015). We analyzed and compared the immune cell fractions in tumor and adjacent non-tumor tissues of ESCC patients employing the “CIBERSORT” and “ggpubr” R packages. Correlations between chemoradiotherapy sensitivity-related genes and immune cell fractions were examined using the “corrplot” R package.

### Associations between key genes and immunoregulatory genes

To explore the associations between chemoradiotherapy sensitivity-related genes and immunoregulatory genes, we sourced gene encodings for immunomodulators from the TISIDB database (Ru et al., 2019), including 24 immunoinhibitors and 46 immunostimulators. After intersecting these with the ESCC gene expression profile, 60 genes encoding immunomodulators were retained. Pearson correlation analysis was conducted on the expression of immunoregulatory genes and chemoradiotherapy sensitivity-related genes. *P* < 0.05 was deemed statistically significant.

### Drug sensitivity prediction

The Genomics of Drug Sensitivity in Cancer (GDSC) database records the responsiveness of cancer cells to drugs and molecular markers associated with drug response (Yang et al., 2013). Utilizing this pharmacogenomics database, the “oncoPredict” R package assessed the half-maximal inhibitory concentration (IC50) of chemotherapeutics for ESCC patients. We evaluated the impact of chemoradiotherapy sensitivity-related gene expression on chemotherapy sensitivity in these patients.

### Expressions of key genes in pan-cancer

Employing the GEPIA database (http://gepia.cancer-pku.cn), we analyzed the differential expression of key chemoradiotherapy sensitivity-related genes in tumor samples and paired normal tissues across 32 cancer types, including ESCC.

### Construction and assessment of nomogram

Cox regression analyses were performed on clinical factors to identify independent prognostic indicators. We constructed a nomogram incorporating these prognostic factors and chemoradiotherapy sensitivity-related genes using the “rms” R package, estimating the 1-, 3-, and 5-year survival probabilities for ESCC patients. The nomogram’s consistency and accuracy were validated with a calibration curve.

### Functional enrichment analysis

Using the “fgsea” and “enrichplot” R packages and the annotated gene set ‘h.all.v2023.2.Hs.symbols.gmt’ from the Molecular Signatures Database, we performed fast gene set enrichment analysis (fGSEA) to identify potential pathways and biological functions differing among chemoradiotherapy sensitivity-related gene expression groups. Based on the Net enrichment score (NES) and *P-*value, we identified significantly enriched hallmark pathways and explored the mechanisms through which key genes influence these pathways.

### Genome-wide association study (GWAS)

Utilizing data from 452,264 individuals in the United Kingdom Biobank and documented in the Gene Atlas database (http://geneatlas.roslin.ed.ac.uk/), which links 778 traits to 30 million variants, we pinpointed pathogenic regions associated with key genes by analyzing GWAS data.

### Statistical analysis

Statistical analyses were conducted using R software (version 4.3.1) (https://www.r-project.org). Survival analysis was performed using the Kaplan-Meier method and comparisons were made using the log-rank test. Pearson correlation tests evaluated relationships between variables. *P* < 0.05 was considered statistically significant.

## Results

### Identification of DEGs between pCR and npCR patients in ESCC

**FIGURE 1:**
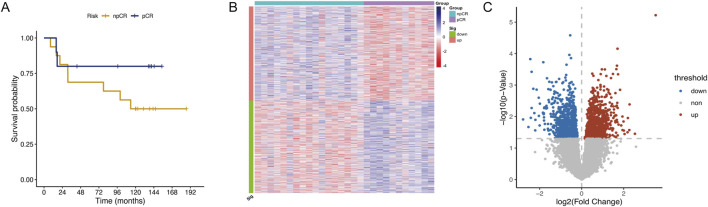
Identification of DEGs between pCR and npCR patients in ESCC. **(A)** Kaplan-Meier curve of pCR (n = 10) and npCR (n = 16) ESCC patients in the Guangzhou cohort. The heatmap **(B)** and volcano plot **(C)** of DEGs between pCR and npCR ESCC patients.

The Guangzhou cohort comprised 28 ESCC patients, including 39.3% (11/28) achieving pCR and 60.7% (17/28) exhibiting npCR. The clinicopathological features of these patients are summarized in Supplementary Table 1. Survival data for patients who underwent NRCT and surgical resection and survived at least 3 months after treatment was collected and performed Kaplan-Meier analysis to evaluate their survival probabilities in both groups. Patients who achieved pCR demonstrated a higher survival probability than those with npCR, particularly after 10 years (Figure 1A, n = 26). However, due to the limited sample size, the differences between the groups were not statistically significant. To identify key chemoradiotherapy sensitivity-related genes, we analyzed DEGs between pCR and npCR patients. We identified 1,726 DEGs, with 870 genes upregulated and 856 genes downregulated in npCR patients. The heatmap and volcano plot illustrating these DEGs are presented in Figures 1B, C.

### Identification of key genes in ESCC

**FIGURE 2:**
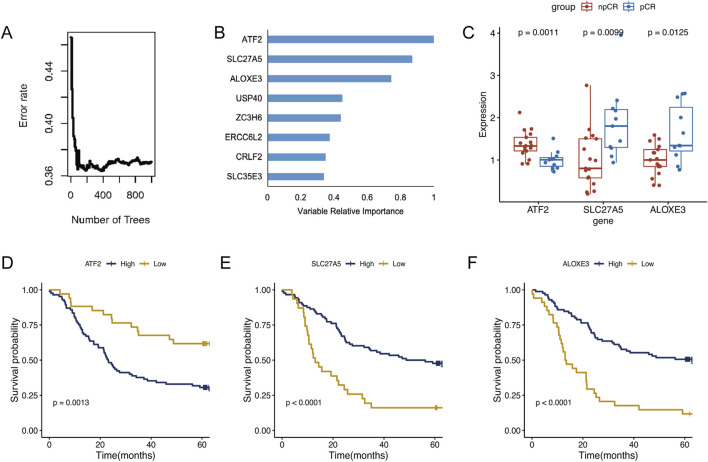
Identification of key genes in ESCC. **(A)** The random survival forest analysis of chemoradiotherapy sensitivity-related genes in ESCC. **(B)** The variable relative importance of three genes >0.7 were identified as key genes in ESCC. **(C)** Expression levels of *ATF2*, *SLC27A5*, and *ALOXE3* in pCR and npCR patients. **(D–F)** Kaplan-Meier survival analysis of ESCC patients with high- and low-expression of *ATF2*, *SLC27A5*, and *ALOXE3*.

To further identify key chemoradiotherapy sensitivity-related genes in ESCC, we conducted univariate Cox regression analysis using RNA-seq and prognostic data from the Beijing Cohort (Supplementary Table 1, n = 119). This analysis identified 126 prognosis-associated protein-encoding genes, comprising 52 associated with a good prognosis and 74 indicating a poor prognosis. After selecting genes highly expressed in pCR patients that indicate a good prognosis and genes lowly expressed in pCR patients indicating a poor prognosis, we identified 35 favorable and 40 unfavorable genes. Following random survival forest analysis, genes with a relative importance >0.7 were deemed key chemoradiotherapy sensitivity-related genes in ESCC. Ultimately, three genes, *ATF2*, *SLC27A5*, and *ALOXE3,* met the screening criteria and are illustrated in Figures 2A, B. Among these, *SLC27A5* and *ALOXE3* were not only highly expressed in pCR patients (*P* < 0.01 and *P* = 0.01, Figure 2C), but also significantly correlated with a favorable prognosis of ESCC (*P* < 0.001, Figures 2E, F). However, ATF2 was highly expressed in npCR patients (*P* = 0.001, Figure 2C) and was significantly associated with poor prognosis (*P* = 0.001, Figure 2D). By employing the X-tile software, the optimal expression level cut-off values of three genes were determined to be 12.5, 10.63 and 7.89, respectively.

By expanding the real-world sample (n = 50), we reanalyzed RNA-seq data from ESCC patients who underwent NCRT followed by surgery. Overall, their mean age was 58.2 years, 43 (86%) were male, 7 (14%) were female, 14 (28%) had stage II disease, and 36 (72%) had stage III disease. The expression of *ATF* in npCR patients was significantly upregulated compared with that in pCR patients, while the expression of *SLC27A5* and *ALOXE3* was significantly downregulated (Supplementary Figure 1A, B), which was consistent with our previous results.

### Immune cell infiltration in ESCC

**FIGURE 3:**
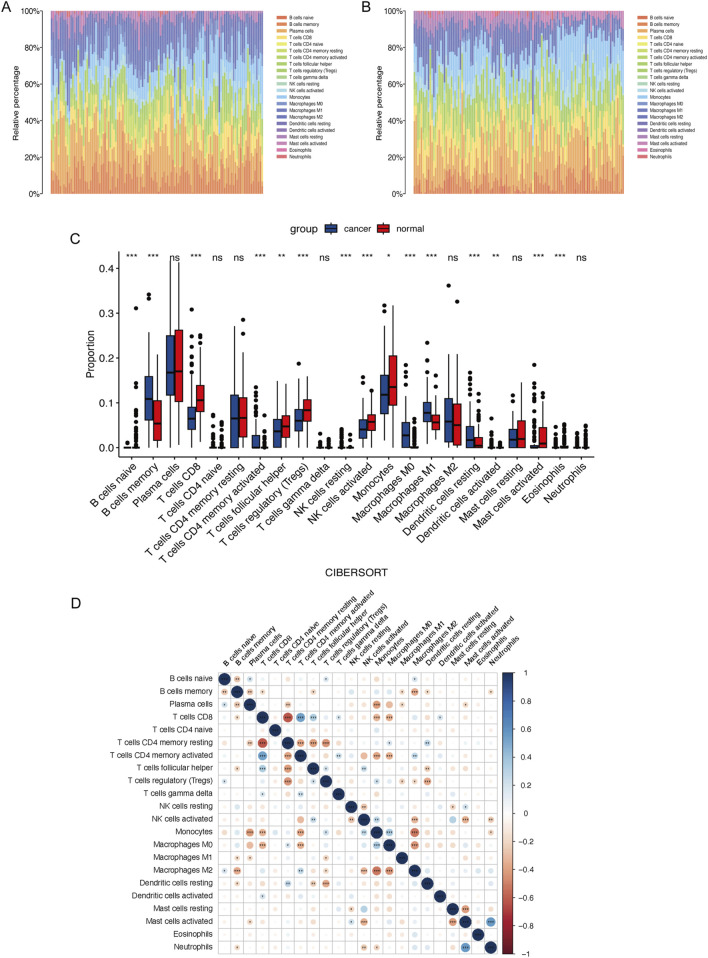
Immune cell infiltration in ESCC. Percentage of 22 types of immune cells infiltration in the cancer **(A)** and normal tissue **(B)** in ESCC patients. **(C)** Comparison of the immune cells proportion between cancer and normal tissue in ESCC patients. **(D)** Correlations between immune cells in ESCC.

**FIGURE 4:**
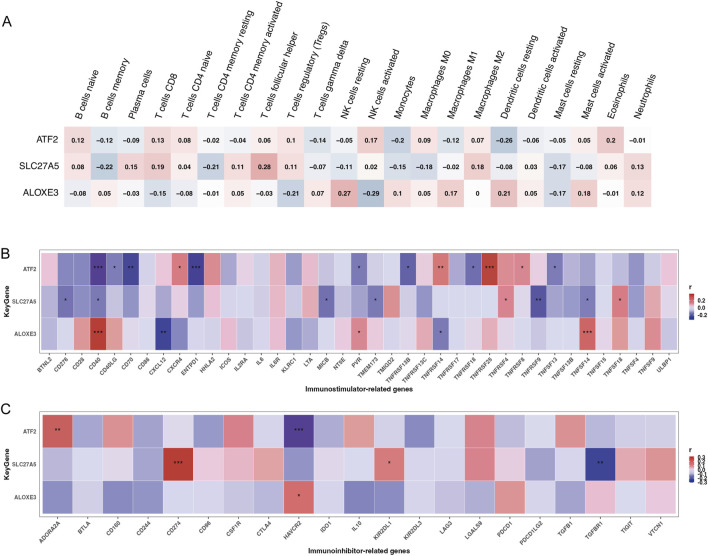
The relationship between key genes and immune cell infiltration and immunoregulators. The relationship of chemoradiotherapy sensitivity-related genes and infiltrating immune cells **(A)**, immunostimulators **(B)**, and immunoinhibitors **(C)** (*P < 0.05,**P < 0.01,***P < 0.001; red color represented positive correlation, blue color represented negative correlation; darker colors indicated stronger correlations).

We analyzed immune cell infiltration between cancerous and normal tissues to assess immunological variations in ESCC. The proportions of infiltrating immune cells in each group were displayed in Figures 3A, B. Compared to normal tissue, cancerous tissue in ESCC showed increased infiltration of memory B cells, activated memory CD4^+^ T cells, resting NK cells, M0 macrophages, M1 macrophages, resting dendritic cells, and activated dendritic cells and decreased infiltration of naive B cells, CD8^+^ T cells, follicular helper T cells, regulatory T cells, activated NK cells, monocytes, activated mast cells, and eosinophils (Figure 3C). Correlations between infiltrating immune cells in ESCC were illustrated in Figure 3D. Further analysis of the associations between the expression of the three key genes and tumor immune cell infiltration revealed that *ATF2* negatively correlated with the infiltration of memory B cells, monocytes, and resting dendritic cells, among others. *SLC27A5* negatively correlated with the infiltration of memory B cells, resting memory CD4^+^ T cells, and M0 macrophages, among others. *ALOXE3* negatively correlated with the infiltration of regulatory T cells, activated NK cells, and resting mast cells, among others (Figure 4A).

### Associations between key genes and immunoregulatory genes

Considering that cancer patients often exhibit immune abnormalities related to tumor immune escape mechanisms, and the necessity to tailor immunotherapy targets and strategies based on individual immune characteristics (Sadun et al., 2007), we explored the associations between three key genes and immunoregulators, including 24 genes encoding immunoinhibitors and 46 genes encoding immunostimulators. Results indicated that *ATF2* was significantly positively correlated with *TNFRSF25*, *TNFRSF14*, and *ADORA2A,* and negatively correlated with *CD40*, *CD70*, *ENTPD1*, and *HAVCR2*. *SLC27A5* was significantly positively correlated with *CD274,* and negatively correlated with *TNFRSF9* and *TGFBR1*. *ALOXE3* was significantly positively correlated with *CD40* and *TNFSF14,* and negatively correlated with *CXCL12* (Figures 4B, C, all *p* < 0.01).

### Evaluation of drug-sensitivity prediction ability of key genes

**FIGURE 5:**
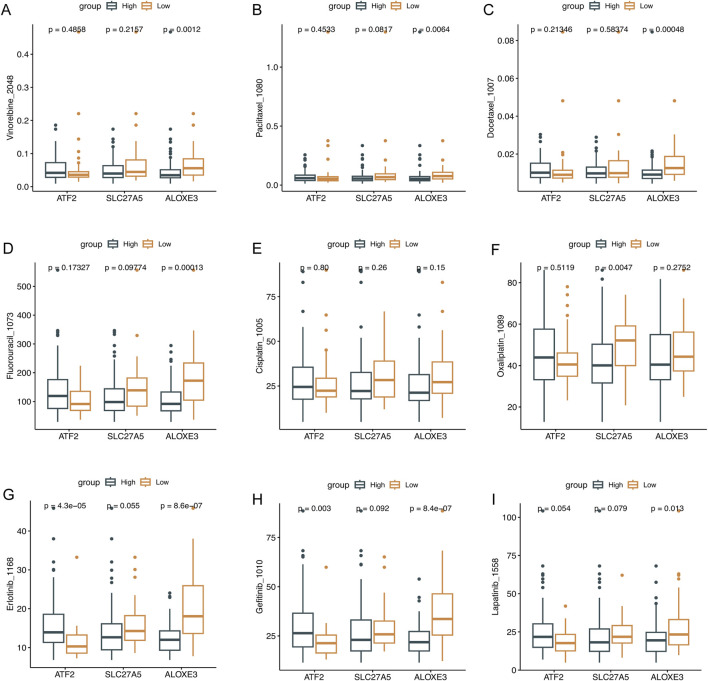
The evaluation of drug sensitivity. Drug sensitivity analysis of the low- and high-expression of *ATF2*, *SLC27A5*, and *ALOXE3* groups. **(A)** Vinorelbine, **(B)** Paclitaxel, **(C)** Docetaxel, **(D)** Fluorouracil, **(E)** Cisplatin, **(F)** Oxaliplatin, **(G)** Erlotinib, **(H)** Gefitinib, and **(I)** Lapatinib.

The “pRRophetic” R package assessed the potential of key genes to predict drug sensitivity in ESCC patients. Compared with patients exhibiting higher expression of *ATF2* and lower expression of *SLC27A5* and *ALOXE3*, IC50 values for drugs including vinorelbine, paclitaxel, docetaxel, fluorouracil, cisplatin, oxaliplatin, erlotinib, gefitinib, and lapatinib, were lower in patients with decreased expression of *ATF2* and increased expression of *SLC27A5* and *ALOXE3*. Specifically, patients with higher *ATF2* expression were significantly less responsive to erlotinib and gefitinib, whereas those with increased *ALOXE3* expression were significantly more sensitive to vinorelbine, paclitaxel, docetaxel, fluorouracil, erlotinib, gefitinib, and lapatinib. Patients with higher *SLC27A5* expression were more sensitive to oxaliplatin (Figures 5A–I, *P* < 0.05). This further confirmed the clinical utility of these key genes in ESCC patients.

### Expressions of key genes in pan-cancer

Using the GEPIA database (http://gepia.cancer-pku.cn), we analyzed the differential expression of key chemoradiotherapy sensitivity-related genes in tumor samples and paired normal tissues across 32 cancers, including ESCC. The results showed that *ATF2* is highly expressed in the tumor tissues of diffuse large B-cell lymphoma, esophageal carcinoma, pancreatic adenocarcinoma, stomach adenocarcinoma, and thymoma compared to normal tissues (Supplementary Figure 2A). However, the expressions of *SLC27A5* and *ALOXE3* were not significantly upregulated in esophageal cancer tissues compared to control tissues (Supplementary Figure 2B, C).

### Construction and assessment of nomogram

**FIGURE 6:**
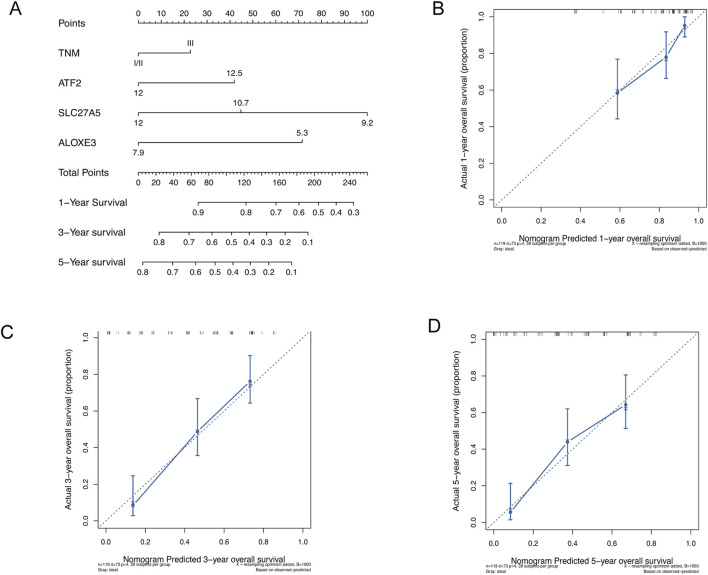
Construction and assessment of nomogram. **(A)** Construction of nomogram based on TNM staging and the expression of *ATF2*, *SLC27A5*, and *ALOXE3*. **(B–D)** Calibration curves of nomogram for OS prediction at 1-, 3- and 5- year in ESCC.

We conducted Cox regression analyses on clinical factors to identify independent prognostic factors. Due to the limited number of TNM stage I cases (n = 6) in the Beijing cohort, we combined stages I and II for analysis. The nomogram was then constructed based on TNM staging and the expression levels of *ATF2*, *SLC27A5*, and *ALOXE3* to quantitatively predict 1-, 3-, and 5-year survival probabilities, providing a reference for clinical decision-making in ESCC patients. The results showed that all three chemoradiotherapy sensitivity-related genes had a greater impact on prognosis prediction than TNM staging, with *SLC27A5* contributing the most (Figure 6A). Calibration curves for 1-, 3- and 5-year OS demonstrated high consistency between the predictions and actual observations, underscoring the prognostic predictive power of these genes in ESCC (Figures 6B–D).

### Gene set enrichment analysis (GSEA)

Utilizing hallmark gene sets from the Molecular Signatures Database, we conducted fast gene set enrichment analysis (fGSEA) to discern the differences in biological processes between high and low expressions of key genes. For the high-expression ATF2 group, the top three upregulated pathways were epithelial-mesenchymal transition (EMT), mitotic spindle, and myogenesis, while the top three downregulated pathways were oxidative phosphorylation, fatty acid metabolism, and KRAS signaling DN. Additionally, the p53 pathway was also downregulated (Supplementary Figure 3A, B). Conversely, the high-expression SLC27A5 group showed downregulation in the EMT pathway (Supplementary Figure 3C, D). Moreover, compared to the high-expression ATF2 group, the high-expression ALOXE3 group exhibited increased activity in the p53 pathway and the EMT pathway (Supplementary Figure 3E, F). These findings suggest that chemoradiotherapy sensitivity-related genes may influence ESCC progression by regulating the epithelial-mesenchymal transition and P53 pathways.

### GWAS analysis

Using GWAS data, we identified the pathogenic regions of key genes in esophageal cancer (Supplementary Figure 4A, B). *ATF2*, *SLC27A5*, and *ALOXE3* were found in the pathogenic regions of chromosomes 2, 19, and 17, respectively (Supplementary Figure 4C–E).

## Discussion

The high mortality rate of ESCC is associated with delayed diagnosis, tumor metastasis, treatment resistance, and recurrence (Sung et al., 2021; Thakur et al., 2021; Puhr et al., 2023). Owing to the advanced stage at diagnosis, nearly 50% of patients are not eligible for complete surgical resection. Evidence from several large clinical trials suggest that NCRT combined with esophagectomy is more effective than surgical resection alone (van Hagen et al., 2012; Yang et al., 2021a). However, due to tumor heterogeneity, therapeutic outcomes vary significantly among patients. Exploring the mechanisms of chemoradiotherapy sensitivity is crucial for improving ESCC prognosis. In this study, we investigated biomarkers and potential mechanisms of chemoradiotherapy sensitivity in ESCC patients. The findings could inform the development of precise treatment strategies for ESCC based on tumor molecular heterogeneity.

Our study revealed that *ATF2* is not only linked to chemoradiotherapy insensitivity in ESCC, but also indicative of poor prognosis. Located on chromosome 2q32 (Ozawa et al., 1991), *ATF2* has been shown in various cancer models to be overexpressed, phosphorylated, and mislocalized subcellularly. It interacts with oncogenic proteins such as JUN (Lopez-Bergami et al., 2010). Activation of the Rac1-P38-ATF2 signaling pathway in non-small cell lung cancer cells has been documented to upregulate the expressions of Cyclin A2, Cyclin D1, and MMP2 proteins, thus promoting tumor growth (Zhou et al., 2018). *ATF2* also binds to the promoter of miR-3913-5p, negatively regulating its expression, which targets CREB5 directly. Overexpression of *ATF2* enhances growth, migration, and invasion in colorectal cancer cells through this mechanism. The ATF2/miR-3913-5p/CREB5 axis is considered a potential therapeutic target for colorectal cancer (Dai et al., 2023). Furthermore, *ATF2* upregulation in gastric cancer correlates with a worse clinical prognosis, and silencing *ATF2* suppresses the malignant phenotype of gastric cancer cells. Notably, reducing *ATF2* expression significantly increases the sensitivity to sorafenib therapy (Xu et al., 2023a). Additionally, *ATF2* is implicated in platinum resistance in non-small cell lung cancer treatment and is targeted to restore chemotherapy sensitivity to platinum. Studies have confirmed that triptolide can enhance chemotherapy efficacy in drug-resistant cell lines by inhibiting the ATF2/cJUN function (Lo et al., 2015).

*SLC27A5* encodes fatty acid transport protein 5. Reduced expression of *SLC27A5* is closely associated with poorer OS in ovarian cancer patients (Chen et al., 2021). Moreover, expression levels of *SLC27A5* are significantly lower in patients with hepatocellular carcinoma (Wang et al., 2022). *SLC27A5* suppresses proliferation and migration of hepatocellular carcinoma cells *in vitro*. This effect is likely due to *SLC27A5*’s role in promoting cuproptosis in hepatocellular carcinoma via upregulation of *FDX1*, making it a potential target for cuproptosis induction in this cancer type (Li et al., 2023). Critically, SLC27A5 enhances the therapeutic efficacy of sorafenib in hepatocellular carcinoma by promoting sorafenib-induced ferroptosis through inhibition of the NRF2/GSR pathway. A deficiency in SLC27A5 correlates with resistance to sorafenib in these cells (Xu et al., 2023b). Additionally, the overexpression of *SLC27A5* curtails the activation of KEAP1/NRF2 pathway and reduces TXNRD1 expression, which can augment the antitumor activity of sorafenib either genetically or pharmacologically by inhibiting the NRF2/TXNRD1 pathway (Gao et al., 2020).

*ALOXE3* is a member of the mammalian lipoxygenase family, playing a role in lipid metabolism and acting as a regulator of ferroptosis (Yang et al., 2016; Yamamoto, 1992). In our study, high expression of *ALOXE3* was linked to improved prognosis in ESCC and increased sensitivity to chemoradiotherapy. Compared to normal human brain tissue, *ALOXE3* expression is significantly diminished in glioblastoma tissue. *ALOXE3* deficiency not only enhances the survival and migration of cancer cells but also supports glioblastoma growth in immunodeficient nude mice. It is crucial for p53-mediated ferroptosis, with miR-18a downregulating its expression by targeting ALOXE3 directly, thus contributing to resistance against p53-induced ferroptosis in glioblastoma cells (Yang et al., 2021b). Transcriptional upregulation of ALOXE3 by YAP promotes lipid peroxide accumulation and induces ferroptosis, making the YAP-ALOXE3 signaling pathway a potential biomarker for predicting ferroptosis-induced responses in hepatocellular carcinoma cells (Qin et al., 2021). In breast cancer, SBFI26, an inhibitor of FABP5, induces ferroptosis by elevating levels of ferrous ions and lipid peroxidation, increasing the expression of *ALOXE3*, *ALOX5,* and *ALOX15*, thus driving ferroptosis in tumor cells (He et al., 2024). Furthermore, the novel ferroptosis inducer, talaroconvolutin A, has been shown to trigger ferroptosis and suppress colorectal cancer cell growth by modulating *ALOXE3* expression and other genes (Xia et al., 2020).

In a previous clinical study, triprilizumab combined with NCRT and surgical resection was used to treat locally advanced ESCC, achieving pCR rate of 50% and an R0 resection rate of 98% (Chen et al., 2023). EC-CRT-001, a prospective clinical trial, enrolled patients with unresectable ESCC and treated them with concurrent chemotherapy, radiotherapy, and triplizumab immunotherapy. Results showed that 62% of these patients achieved a complete response, with a median response duration exceeding 1 year, and a 1-year OS rate of 78.4%. The combination of immunotherapy with definitive chemoradiotherapy offers a promising treatment strategy for patients with unresectable, locally advanced ESCC (Zhu et al., 2023). However, the potential synergistic mechanism between immunotherapy and chemoradiotherapy remains unclear. Our results indicated that key genes associated with chemoradiotherapy sensitivity were significantly correlated with immune cells and immunomodulatory genes, suggesting a potential mechanism for their synergistic effect in ESCC.

In our study, the EMT pathway was the most significantly upregulated pathway in the high-expression *ATF2* group, and the most significantly down-regulated pathway in the high-expression *SLC27A5* group. EMT is recognized as a complex and coordinated process crucial for cancer initiation and progression (Dongre and Weinberg, 2019). ATF2 binds to the promoter region of GLUT3, enhancing EMT in colorectal cancer by inducing GLUT3 expression (Song et al., 2022). Through promoter binding, ATF2 and endoplasmic reticulum stress induce the expression of CAP2, promoting EMT in liver cancer cells (Yoon et al., 2021). In conjunction with TGF-beta1, ATF2 induces EMT in pancreatic cancer cell lines (Xu et al., 2012). Additionally, via the c-Met/BVR/ATF-2 pathway, lncRNA NR2F2-AS1 activates the EMT process and fosters the development of non-small cell lung cancer (Liu et al., 2021). EMT increases malignancy in non-small cell lung cancer cells and reduces chemosensitivity to cisplatin and paclitaxel. EMT marker expression is elevated in cells chronically exposed to these agents or radiation, linking EMT to chemoradiotherapy resistance (Shintani et al., 2011). Similarly, EGFR activation triggers EMT, reducing cellular responsiveness to radiation and cetuximab in head and neck cancer (Holz et al., 2011). Inhibitors of the EMT signaling pathway may enhance the sensitivity of cancer cells to chemoradiotherapy (Shintani et al., 2011). Downregulation of Delta-like Ligand 4 inhibits EMT in cervical cancer, thereby enhancing radiosensitivity (Yang et al., 2020). The EMT signaling pathway could provide a potential direction for further exploration into the mechanism of chemoradiotherapy sensitivity genes in ESCC.

In this study, we evaluated the role of chemoradiotherapy sensitivity-related genes in ESCC through survival analysis, drug sensitivity analysis, and the development and validation of a nomogram. While our results underscored the potential clinical relevance of three key genes in ESCC, there are notable limitations. This was a retrospective study that did not delve into the underlying mechanisms. Future studies should involve independent prospective cohorts and both *in vitro* and *in vivo* experiments to confirm these findings and further explore the mechanisms. Additionally, although these three key genes can predict the response of ESCC to NCRT and are significantly associated with certain immune cells and immunoregulatory genes in the tumor microenvironment, their efficacy in ESCC treated with combined immunotherapy and NCRT remains to be established.

## Conclusion

In conclusion, we identified three pivotal genes that are crucial in predicting both the sensitivity of ESCC to radiochemotherapy and the prognosis of patients. We investigated the associations between these genes and infiltrating immune cells and immunoregulatory genes. Furthermore, we explored the signaling pathways influenced by these genes in ESCC and analyzed the chromosomal regions implicated in their pathogenesis. The identification of these key genes may facilitate the optimization of individual treatment strategies and improve prognosis management in ESCC.

## Data Availability

The datasets presented in this study can be found in online repositories. The names of the repository/repositories and accession number(s) can be found in the article/Supplementary Material.
