# Supplementary figures and images for: Identification of key genes to predict response to chemoradiotherapy and prognosis in esophageal squamous cell carcinoma
Source: Front Mol Biosci. 2024 Nov 20;11:1512715. doi: 10.3389/fmolb.2024.1512715 (PMC11614722; doi:10.3389/fmolb.2024.1512715)

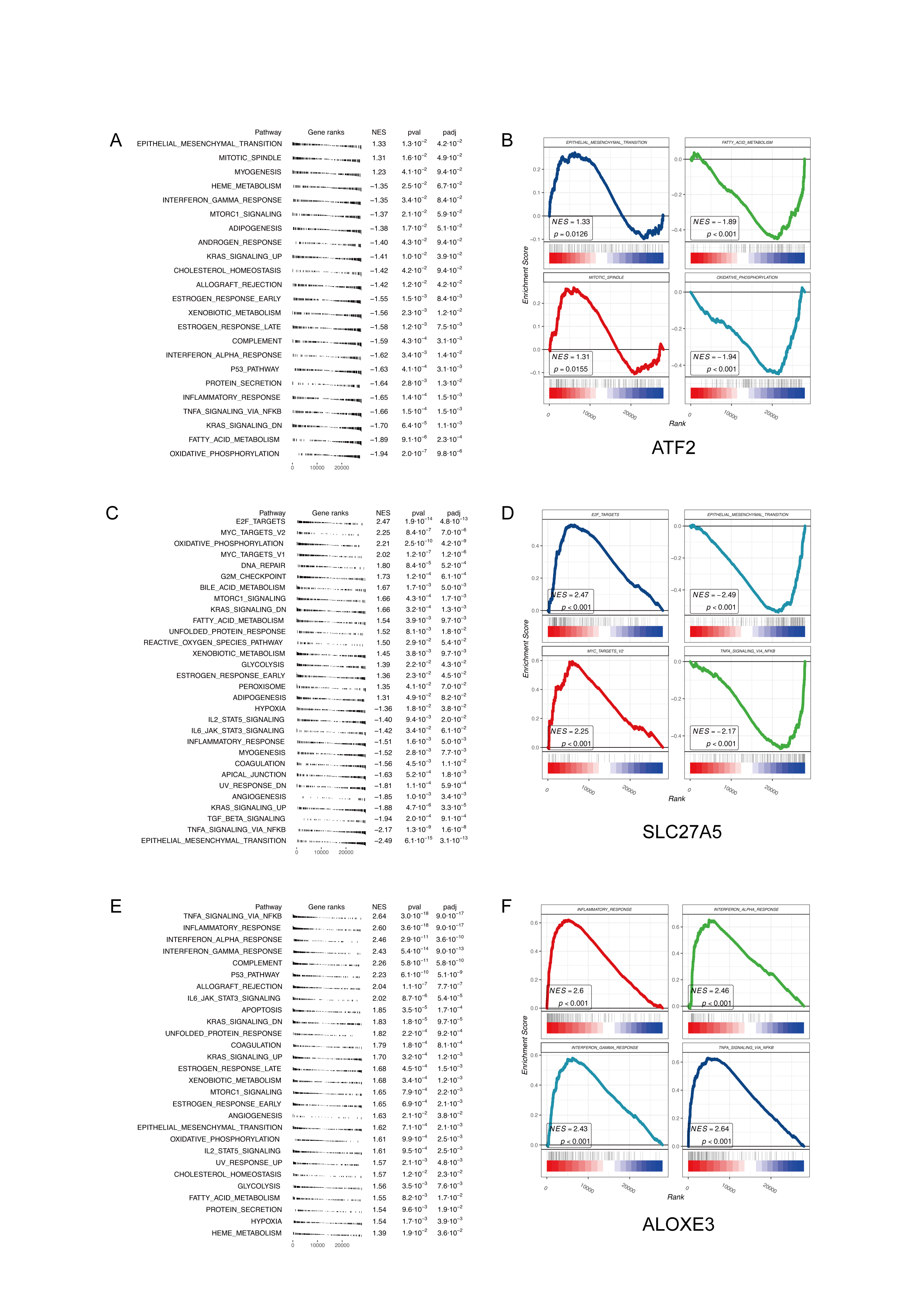

Supplement: Supplementary file 1 [file Image3.tif]

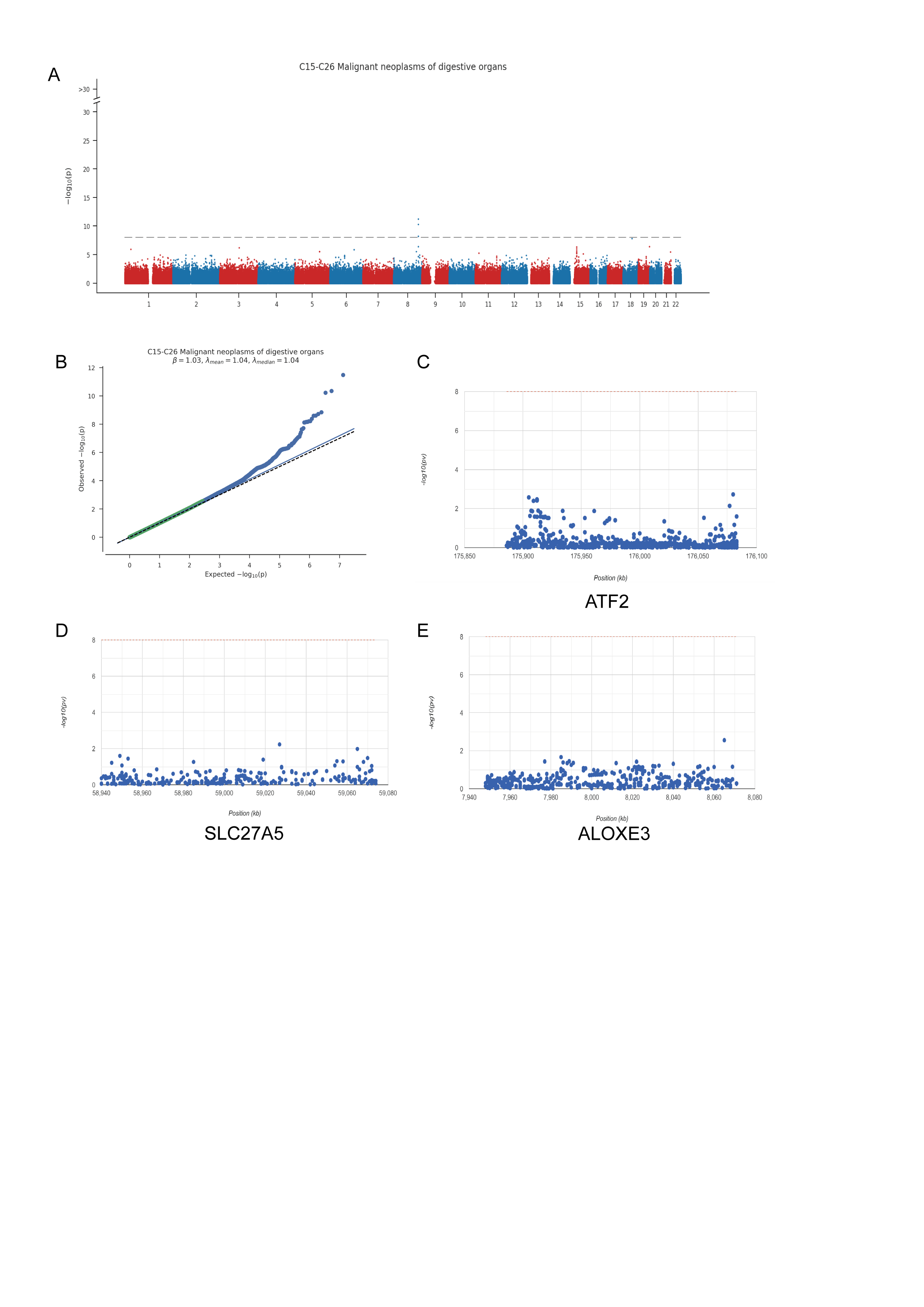

Supplement: Supplementary file 2 [file Image4.tif]

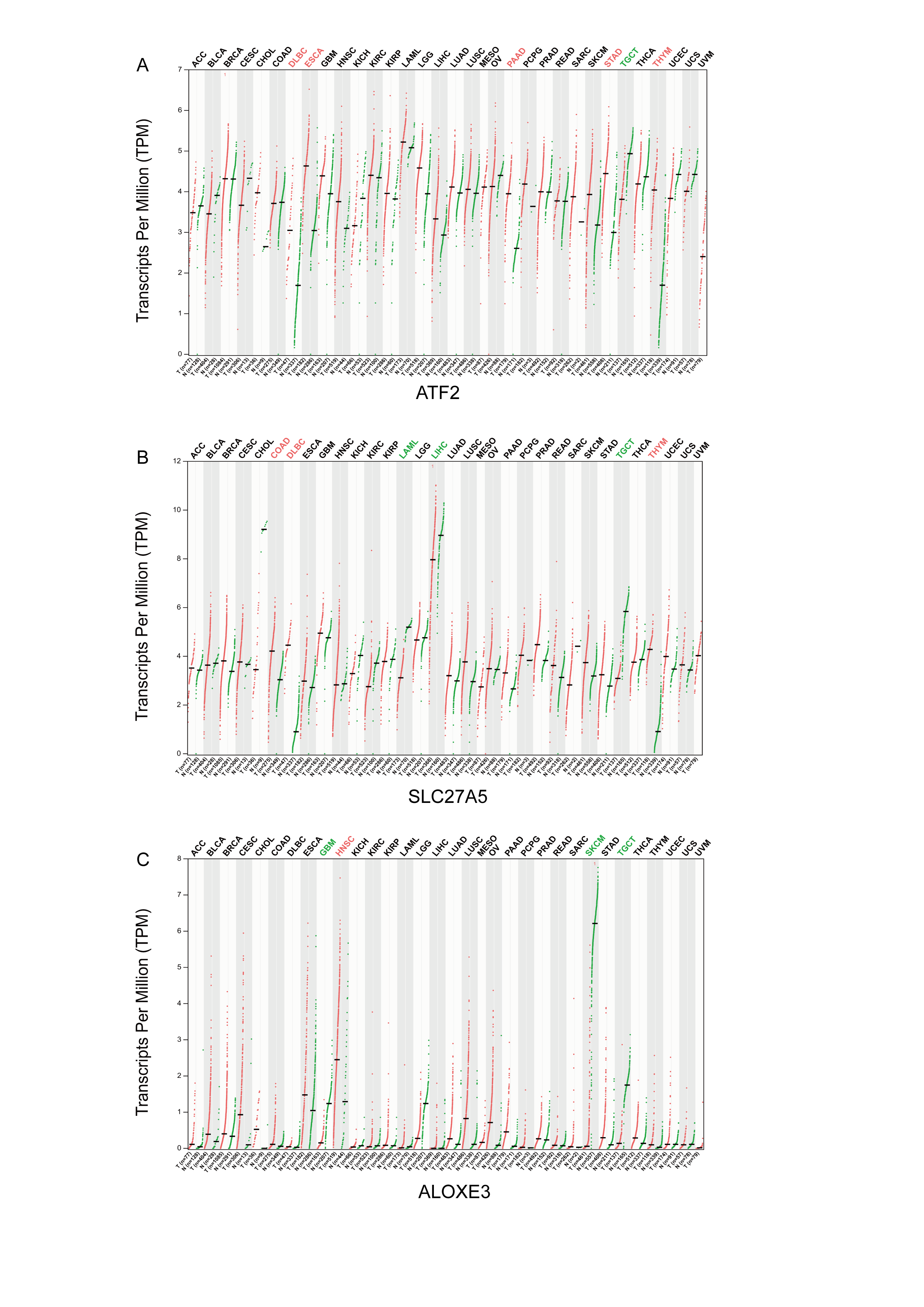

Supplement: Supplementary file 3 [file Image2.tif]

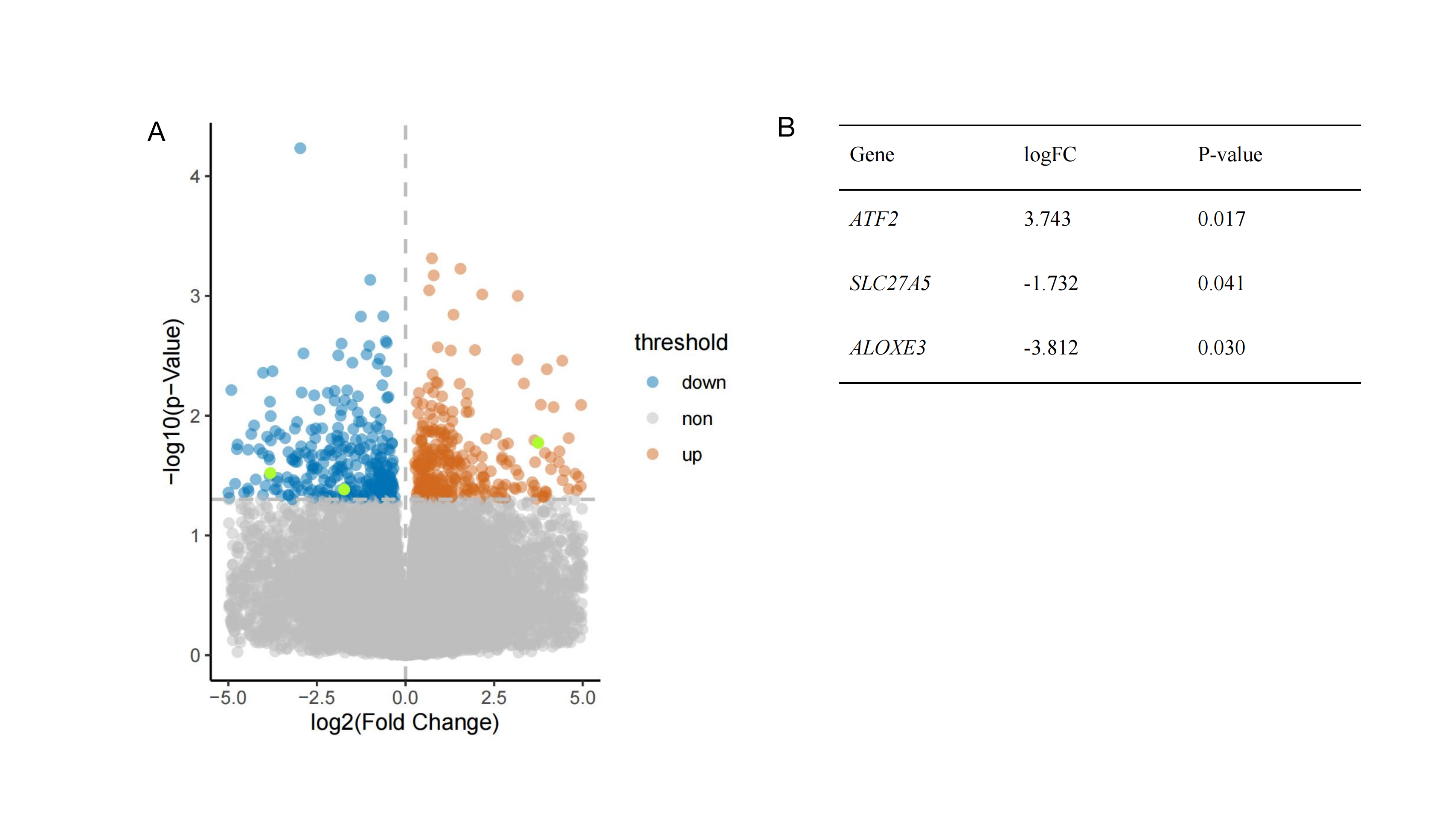

Supplement: Supplementary file 4 [file Image1.tif]
