# Supplementary material for: Identification of key genes to predict response to chemoradiotherapy and prognosis in esophageal squamous cell carcinoma
Source: Front Mol Biosci. 2024 Nov 20;11:1512715. doi: 10.3389/fmolb.2024.1512715 (PMC11614722; doi:10.3389/fmolb.2024.1512715)
Supplement: Supplementary file 5 [file DataSheet1.docx]

Supplementary Table 1. Clinicopathologic characteristics of patients with esophageal squamous cell carcinomas in Guangzhou cohort and Beijing cohort.

| **Variables** | **Guangzhou cohort** | **Beijing cohort** |
| --- | --- | --- |
| Gender |  |  |
| Male | 25 | 98 |
| Female | 3 | 21 |
| Age (years) |  |  |
| <60 | 20 | 61 |
| ≥60 | 8 | 58 |
| Tumor differentiation |  |  |
| Well | 7 | 23 |
| Moderate | 16 | 64 |
| Poor | 5 | 32 |
| T stage |  |  |
| T1 | 0 | 8 |
| T2 | 8 | 20 |
| T3 | 20 | 62 |
| T4 | 0 | 29 |
| N stage |  |  |
| N0 | 0 | 54 |
| N1 | 28 | 42 |
| N2 | 0 | 13 |
| N3 | 0 | 10 |
| M stage |  |  |
| M0 | 28 | 119 |
| M1 | 0 | 0 |
| Response |  |  |
| pCR | 11 | - |
| npCR | 17 | - |

**Supplementary Figure Legend**

**Supplementary Figure 1.** The volcano plot of DEGs between pCR (n=20) and npCR (n=30) real-world ESCC patients (A). The change of expression and p-values of three key genes between pCR and npCR real-world ESCC patients (green dot in A and B).

**Supplementary Figure 2.** Expressions of key genes in pan-cancer. The expression of *ATF2* is significantly increased in esophageal carcinoma (A), while the expression *SLC27A5* (B) and *ALOXE3* (C) shows no significant difference in the tumor and paired normal tissues in esophageal carcinoma.

**Supplementary Figure 3.**GSEA plots based on fgsea algorithm of up- or down-regulated pathways between high- and low-expression of *ATF2* (A and B), *SLC27A5* (C and D), and *ALOXE3* (E and F) groups. (NES: normalized enrichment score; pval: p value; padj: adjusted p value)

**Supplementary Figure 4.** (A)Manhattan plot of GWAS; (B) quantile-quantile (QQ) plot of GWAS; (C-E) *ATF2*, *SLC27A5*, and *ALOXE3* were located in the pathogenic region of chromosome 2, 19, and 7, respectively.
